# The Effect of Ambient Artificial Intelligence Documentation on Clinician Workload, Efficiency, and Patient Experience in a Multisite Emergency Department Network

**DOI:** 10.1055/a-2939-3038

**Published:** 2026-09-14

**Authors:** Markos G. Kashiouris, Andrew Miner, Sameh Saleh, Matthrew Scripps, Lindsey Stanton, Golda Samuel, Brent Dibble

**Affiliations:** 1 Division of Clinical Informatics 3313 INOVA Health SystemFalls ChurchVirginiaUnited States; 2 Division of Health Informatics 3313 INOVA Health SystemFalls ChurchVirginiaUnited States

**Keywords:** clinical information systems, specific types, patient records, electronic health records and systems, process management tools, clinical decision support, artificial intelligence, sociotechnical aspects of information technology

## Abstract

**Background** Ambient artificial intelligence (AI) documentation systems have emerged as a potential solution to clerical burden in medicine, yet evidence in emergency settings remains limited.

**Objectives** The objective of this study is to evaluate the ambient AI on patient experience, documentation efficiency, clinician workload, and clinical throughput across a large emergency department (ED) network.

**Methods** This is a retrospective, observational cohort study of ambient AI rollout across 14 EDs (May 2024–June 2025). Clinicians applied the tool on a voluntary basis and contributed both AI-assisted and conventional notes. Outcomes included patient-reported experience, active editing time, disposition-to-completion time, copied-forward content, and on-time note completion. Clinician workload was assessed with pre- and postimplementation surveys including the NASA Task Load Index (TLX).

**Results** Ambient AI was used in 8.6% of 315,242 ED clinical notes. AI use was associated with higher top-box ratings for clinician listening (82.0 vs. 76.6%; odds ratio [OR]: 1.39; *p* = 0.003) but not likelihood to recommend (77.6 vs. 75.0%; OR: 1.15; *p* = 0.185). Within-clinician paired analysis showed no difference in active editing time between AI and conventional notes (median difference: +0.17 min; 95% confidence interval [CI]: −1.00 to +1.55; *p* = 0.349), and clinicians typed 722 fewer characters per AI-assisted note. Documentation finalized 3.4 h earlier for admitted and 6.0 h earlier for discharged patients; the disposition-to-completion difference was +5.06 h ( *p* = 0.337), reflecting attenuation of early-adopter’s advantage. AI-assisted notes were half as likely to include copied content (9.4 vs. 18.3%; OR: 0.46; *p* < 0.001). NASA-TLX workload decreased by 40.2 points (95% CI: 30.4–50.1).

**Conclusion** Ambient AI documentation was associated with improved patient-reported listening, earlier note completion, reduced copied-forward content, and lower clinician-perceived cognitive burden, without altering active editing time per note. These findings suggest a scalable tool for reducing clerical burden in high-throughput emergency care, while introducing new responsibilities for clinicians to review, edit, and sign machine-generated notes.

## Background and Significance

Physician burnout in emergency medicine has reached alarming levels, posing risks to clinician well-being, patient safety, and health care system sustainability. Emergency department (ED) clinicians are particularly vulnerable due to the unpredictable nature of their work, high-stakes medical decision-making, and productivity expectations. These factors have contributed to a sizable number of emergency medicine physicians leaving the specialty within 5 years of completing residency. 1 Across all medical specialties, physician turnover and reduced clinical hours related to burnout are estimated to cost the US health care system $4.6 billion annually. 2

Over the past decade (2014–2023), ED visits in the United States have risen significantly, exacerbating clinician workloads. 3 At the same time, patients often present with complex clinical situations and higher acuity as they defer care due to financial or access barriers. 4 This shift requires incremental cognitive workload and meticulous, sometimes defensive, documentation, factors strongly associated with emotional exhaustion. 5 6 Indeed, high clinician workload, measured with tools like the NASA-Task Load Index (TLX), is a concern in clinical informatics as it is linked to a higher risk of medical errors, diagnostic inaccuracies, and degradation of patient safety. 7 8 9

Amid these pressures, artificial intelligence (AI)-driven solutions, particularly ambient clinical documentation technologies, have emerged as promising tools to ease this burden. By automating one of the most time-consuming aspects of patient clinical notes, these technologies may reduce clinician burden and enable behaviors associated with clinical presence and empathy, such as maintaining eye contact. 10 11 12

## Objectives

Our aim was to investigate how the implementation of an ambient clinical documentation tool for emergency clinicians could affect documentation efficiency, clinical throughput, patient experience, and clinician workload.

## Methods

### Study Design and Implementation Timeline

We performed a retrospective, within-clinician observational cohort study of a staggered implementation of an ambient documentation system across 14 EDs in a single integrated health system from May 2024 to June 2025. This observational cohort follows the Strengthening the Reporting of Observational Studies in Epidemiology guidelines. The study was approved by the institutional review board (IRB) number: INOVA-2025-264.

**Fig. 1:**
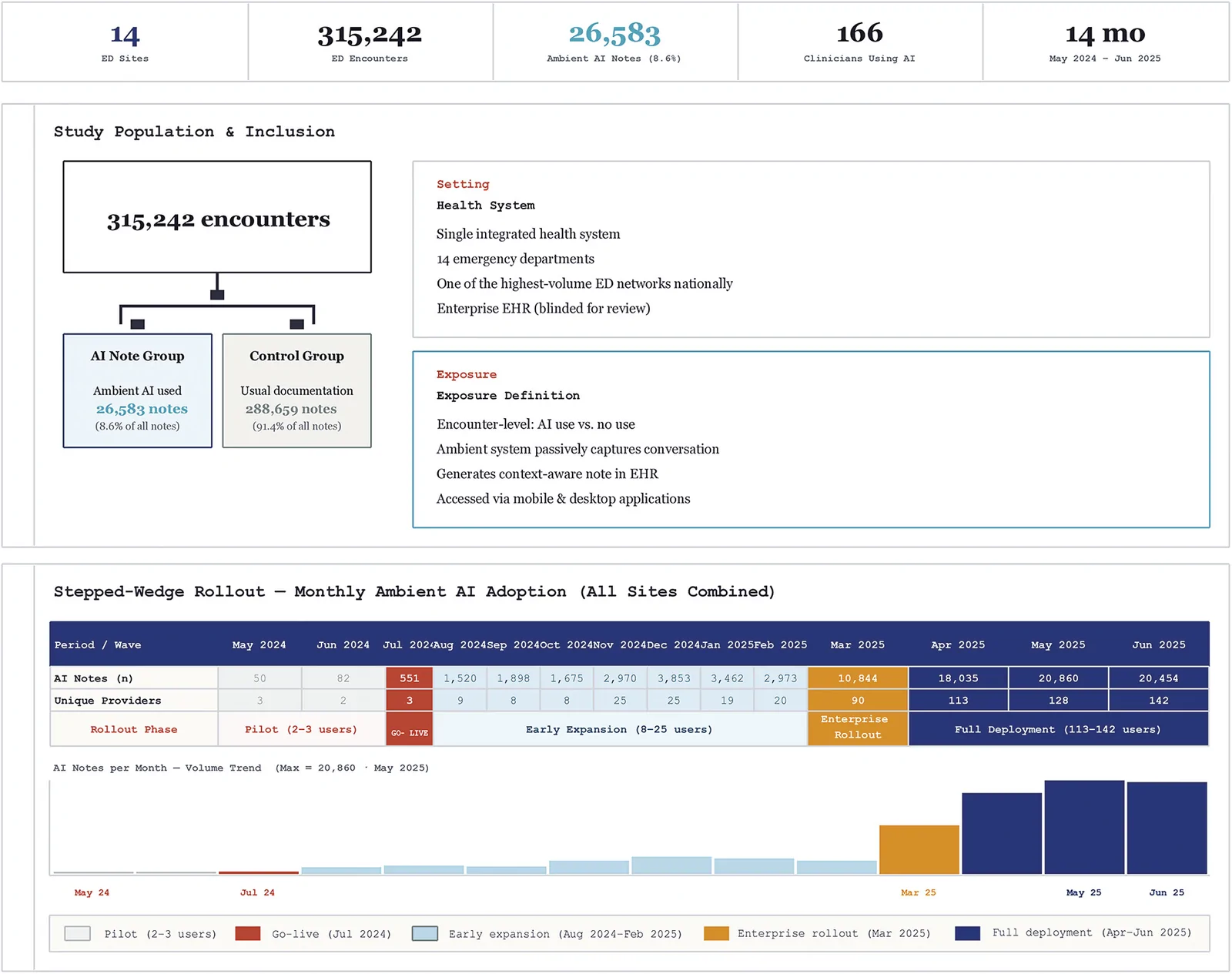
Study design overview: staggered implementation of ambient AI documentation across 14 Inova Health System emergency departments (May 2024–June 2025). AI, artificial intelligence.

The implementation timeline is outlined below and illustrated in Fig. 1 .

- Preenterprise adopter cohort: May 2024 to February 2025.
- Enterprise rollout: March 2025 (the principal rollout step).
- Full deployment: April 2025 onward.

### Participants, Exposure Definition, and Unit of Analysis

All ED clinician-authored notes with valid timestamps during the study period were eligible for inclusion. All ED attending physicians and advanced practice providers, hereafter referred to as *clinicians* , were eligible to enroll for a license and use the ambient documentation system. The study population included all clinicians who used the system at any point during the pilot, early-expansion, or enterprise rollout phases.

A subset of 31 clinicians had early access to the AI scribe system prior to the enterprise rollout (pilot and early-expansion phases). These clinicians constituted the *preenterprise adopter cohort* and were retained in the intervention group for all post-March 2025 analyses. Their inclusion reflects the staggered-rollout structure, in which exposure status is determined by system availability and clinician use rather than by calendar-period assignment.

The unit of analysis was the individual clinician-authored note. Each eligible ED clinical encounter could contain multiple clinician-authored notes; each note was treated as an independent observation and linked to its author via a unique clinician identifier. Exposure was defined at the note level as the binary presence or absence of ambient AI-assisted documentation (1 = ambient AI note; 0 = usual documentation). This classification was derived from postquality control metadata within the analytics warehouse and applied independently to each note, irrespective of other notes within the same encounter.

Because clinicians selectively applied the ambient AI scribe on a note-by-note basis, most contributed both exposed and unexposed notes within the same time periods. The primary efficiency analyses therefore leveraged within-clinician comparisons, contrasting each clinician’s AI-assisted notes with their own usual documentation under similar operational conditions. All regression models accounted for repeated measures using clinician-level clustering of standard errors.

The staggered availability of the ambient documentation system across pilot, early-expansion, and enterprise rollout phases created a staggered-adoption structure in which clinicians transitioned from unexposed to exposed status at different points in time. This design allowed both within-clinician and between-clinician comparisons: clinicians who adopted the system early contributed pre- and postexposure observations, whereas later adopters served as contemporaneous controls until their own transition. Sensitivity analyses, including difference-in-differences (DiD), leveraged the sequential adoption pattern to assess early-adopter effects and secular trends. Table 1 outlines the design elements supporting within-clinician comparison.

**Table 1:** Design elements supporting within-clinician comparison: function and contribution to robust comparisons.

Element of the design	Description in this study	Contribution to staggered-adoption structure	Contribution to robust comparison
Staggered rollout phases (pilot → early expansion → enterprise rollout)	Clinicians gained access at different times from May 2024 to March 2025	Creates sequential “steps” where clusters transition from unexposed to exposed	Allows comparison of clinicians before and after their own adoption while controlling time trends
Preenterprise adopter cohort (31 clinicians)	Used the system before the formal rollout	Represents the earliest adoption step	Provides pre/post data and enables difference-in-differences to quantify early-adopter effects
Enterprise rollout as principal step	The majority of clinicians gained access in March 2025	Major transition point in the rollout	Creates contemporaneous controls among later adopters
Note-level exposure classification	Each note labeled AI vs. non-AI	Exposure varies within each rollout step	Enables within-clinician comparisons under similar conditions
Selective, note-by-note use	Clinicians produce both AI and non-AI notes	Natural crossover within each step.	Each clinician serves as their own control, reducing confounding
Clinician-clustered models	Accounts for repeated measures	Aligns with the clustered, staggered-adoption design	Produces valid inference despite correlated outcomes
Sensitivity analyses (DiD, IPTW)	Explicitly leverage staggered adoption	Models’ sequential treatment timing	Tests robustness to early-adopter bias and secular trends

Abbreviations: AI, artificial intelligence; DiD, difference-in-differences; IPTW, inverse probability of treatment weighting.

Note: The staggered rollout of the ambient AI documentation system across 14 emergency departments allowed clinicians to transition from unexposed to exposed status at different points in time, enabling both within-clinician and between-clinician comparisons.

### Software, Training, and Onboarding Process

The intervention was an ambient scribe documentation system (Abridge AI Inc.) that generates context-aware notes integrated into Epic (Epic Systems Corporation) electronic health record (EHR) and was accessed via mobile and desktop applications. A standardized ED documentation template was released system-wide concurrently with the ambient AI rollout. The prior template varied by site; the updated template restructured three sections, History of Present Illness (HPI), Physical Examination, and Assessment and Plan, to align with the ambient AI output format while leaving all other note sections (e.g., Review of Systems, Medical Decision Making, Orders) unchanged. Importantly, the revised template was available to all ED clinicians regardless of whether they used the ambient AI tool. Clinicians who did not adopt the AI system documented using the same updated clinical note template with conventional manual entry. Because both AI users and nonusers documented within the identical template structure during the postimplementation period, the between-group comparison of AI versus non-AI notes inherently controls for template effects on the three modified sections.

The exposure was defined at the clinical note level as the use versus no use of the AI tool (“usual documentation”). None of the manuscript authors reported a conflict of interest with either corporation. Coauthor G.S. was responsible for the training and onboarding process. The onboarding and training process followed a comprehensive, multimodal strategy designed to promote clinician readiness and consistent system use ( **Supplementary Material** , available in online version only).

### Data Sources and Measurements

Clinician workload surveys were administered 2 weeks of preimplementation and 8 weeks of postimplementation. We extracted encounter-, note-, and clinician-level data from the enterprise analytics warehouse. For patient experience analyses, Press Ganey (PG) ED surveys were linked to clinical notes. The PG scores were measured independently and prospectively by the institution, and measurements were blinded to AI documentation status. PG ED surveys use a 5-point Likert scale (1 = “Very Poor” to 5 = “Very Good”) for each item. We defined “top-box” as the proportion of respondents selecting the highest response category (score = 5), which is the industry-standard metric used by PG for benchmarking and public reporting. The two items analyzed, “Doctors took the time to listen” and “Likelihood to recommend,” are individual survey questions, not composite scales.

Process and efficiency measures were captured from EHR metadata, including manually typed characters and time spent editing. “Editing time” was operationalized identically for both AI-assisted and conventional notes as the cumulative seconds the clinician spent with the note open in active editing mode within the EHR, measured from the first keystroke or cursor placement after the initial draft (whether AI-generated or manually composed) through note signature. For AI-assisted notes, this captures the postdraft review, modification, and finalization of the AI-generated text. For conventional notes, it captures the revision and finalization phase after initial free-text entry or dictation import. The metric excludes idle time (no keyboard or mouse activity for >60 s) and does not include the initial dictation capture or free-text composition phase for either group.

Three distinct documentation-time metrics were used and are reported separately throughout: (1) active editing time, the cumulative minutes with the note open in active editing mode, as defined above (within-clinician analysis, **Supplementary Table S2** , available in the online version only); (2) total documentation time, total note-authoring time per encounter; and (3) disposition-to-completion time, the elapsed hours from patient disposition to note finalization (the DiDs outcome, Table 2 , reported in hours).

**Table 2:** Sensitivity analysis: difference-in-differences decomposition of mean emergency department note completion time (h).

Panel A. primary DiD analysis (full cohort)				
	Preintervention	Postintervention	Difference (post–pre)	*p* -Value
Control group (no AI note)	27.67	24.86	−2.81	
*N* (clinicians/notes)	248/248,634	179/880,063		
Treatment group (AI note)	19.06	21.31	+2.25	
*N* (clinicians/notes)	2–3/205	166/26,583		
Difference (treatment − control)	−8.61	−3.55	DiD = +5.06	0.337 ^a^

Abbreviations: AI, artificial intelligence; DiD, difference-in-differences; ED, emergency department.

Notes: Values are predicted mean hours from ED disposition to note completion, derived from generalized linear models with clinician-clustered robust standard errors, adjusted for confounders present in Table 3 .

Preintervention = April–June 2024 (pilot phase, 2–3 clinicians); postintervention = March–December 2025 (enterprise rollout, 113–178 clinicians/month).

The DiD estimator represents the difference between pre-to-post changes in the treatment and control groups: (+2.25) − (−2.81) = +5.06 h. A positive value indicates the treatment group’s early advantage attenuated relative to controls.

^a^ The DiD estimator of +5.06 h was not statistically significant ( *p* = 0.337). The nonsignificant result reflects the small preimplementation treatment cell (2–3 pilot clinicians vs. 248 controls), yielding insufficient statistical power to detect a differential trend. The preintervention treatment mean (19.06 h) is based on approximately 205 notes from the pilot cohort, whereas the control mean (27.67 h) derives from 248,634 notes, creating a >1000-fold sample-size imbalance.

Pre- and postimplementation clinician surveys included the 3-item short form of the NASA-TLX, assessing mental demand, temporal demand, and effort. The 3-item NASA-TLX (0 to 20) was linearly rescaled to 0 to 100 (×5) to align with common reporting; we acknowledge this does not increase underlying measurement granularity. Surveys were distributed to all active ED clinicians ( *N* = 329); 25 (7.6%) responded preimplementation and 15 (4.6%) postimplementation. Because participation was anonymous, exact respondent overlap could not be linked at the individual level; the demographic and role distributions of the two samples indicated the waves were largely nonoverlapping, and independent-samples *t* tests were therefore used.

We restricted the patient experience analysis to “Doctors took the time to listen” and “Likelihood to recommend the ED clinician” because these were the only two PG items with a plausible causal pathway to ambient AI documentation: the tool captures the clinician–patient conversation, potentially enabling more attentive listening behaviors and greater clinical presence. Other survey items, such as wait times, nursing communication, pain management, and facility ratings, reflect aspects of the ED experience unrelated to the physician documentation workflow and were excluded a priori. We did not analyze other physician-specific items (e.g., “Doctor explained things clearly”) because our prespecified hypothesis centered on listening and recommendation as the outcomes most plausibly affected by passive conversation capture.

Missing data were handled as follows. For EHR-derived documentation metrics (characters typed, editing time, disposition-to-note completion time), encounters with null or implausible values (e.g., negative time intervals, editing time of 0 s for notes containing text) were excluded from the relevant analysis. No imputation was performed. For PG survey data, analyses were restricted to encounters with a completed and linkable survey response; because PG surveys are distributed to a sample of patients and response is voluntary, the effective survey linkage rate represents a fraction of total encounters. For the NASA-TLX clinician workload survey, all respondents who completed the 3-item short form were included ( *n* = 25 preimplementation, *n* = 15 postimplementation); there were no partially completed instruments. A complete-case analytic approach was used throughout.

### Outcomes

The primary outcomes were: (1) patient-reported experience, measured as top-box responses for “Doctors took the time to listen” and “likelihood to recommend the ED clinician”; (2) documentation efficiency, measured by characters manually typed per note and active editing time; and (3) documentation timeliness, measured as the disposition-to-completion time and the odds of meeting on-time completion benchmarks (≤24 h for admitted patients, ≤72 h for discharged patients).

### Statistical Analysis

We used generalized linear models to estimate associations between AI note use and outcomes, with robust Huber–White standard errors clustered at the clinician level to account for repeated measures. Results are reported as odds ratios (ORs) or adjusted mean differences with 95% confidence intervals (CIs). Differences in NASA-TLX scores were assessed using two-tailed, independent-sample *t* tests. We performed sensitivity analyses by estimating DiDs comparing adopters versus nonadopters pre- and postrollout, and inverse probability of treatment weighting (IPTW) with clinician-clustered robust standard errors.

Throughout the manuscript, “ *adjusted* ” refers to estimates from models adjusted for the patient- and encounter-level case-mix covariates presented in Table 3 : patient age, Emergency Severity Index (ESI) acuity level, arrival method, and ED disposition, in addition to clinician-level clustering of standard errors. These covariates were selected a priori as the measured characteristics most plausibly associated with both the clinician’s decision to use ambient documentation and the study outcomes.

**Table 3:** Baseline characteristics of clinicians and emergency department encounters by study group.

Part A: clinician characteristics				
Characteristics	Category	Control, *N* = 163	Ambient AI, *N* = 166	SMD
Clinician years since medical school graduation				
Mean (SD)		15.9 (9.1)	17.1 (8.8)	0.13
	3–5 y	6 (3.7%)	0 (0.0%)	0.28
	5–10 y	30 (18.4%)	24 (14.5%)	0.11
	10–20 y	43 (26.4%)	57 (34.3%)	0.17
	>20 y	32 (19.6%)	36 (21.7%)	0.05
	Unknown	52 (31.9%)	49 (29.5%)	0.05
Clinician note volume during study period (quartile)				
	Bottom quartile	0 (0.0%)	2 (1.2%)	0.16
	Second quartile	7 (4.3%)	10 (6.0%)	0.08
	Third quartile	64 (39.3%)	61 (36.7%)	0.05
	Top quartile	92 (56.4%)	93 (56.0%)	0.01
Clinician gender				
	Female	84 (51.5%)	97 (58.4%)	0.14
	Male	63 (38.7%)	66 (39.8%)	0.02
	Unknown	16 (9.8%)	3 (1.8%)	0.35
Clinician professional degree				
	MD	102 (62.6%)	104 (62.7%)	0.00
	DO	8 (4.9%)	12 (7.2%)	0.10
	PA	28 (17.2%)	28 (16.9%)	0.01
	NP	3 (1.8%)	9 (5.4%)	0.19
	FNP	5 (3.1%)	7 (4.2%)	0.06
	DNP, FNP	0 (0.0%)	1 (0.6%)	0.11
	MD, PhD	1 (0.6%)	2 (1.2%)	0.06
	Unknown	16 (9.8%)	3 (1.8%)	0.35

Abbreviations: AI, artificial intelligence; SD, standard deviation; SMD, standardized mean difference.

Note: Control arm = clinicians who authored notes exclusively without ambient AI ( *N* = 163); Intervention arm = clinicians who used the ambient AI scribe for ≥1 note during the study period ( *N* = 166); together comprising all 329 active clinicians. Years since graduation reported as mean (SD); band percentages exclude Unknown from the denominator. Note-volume quartile thresholds based on the distribution across all 329 clinicians. Unknown gender and degree values are shown explicitly. SMD = absolute standardized mean difference, computed from the reported group proportions (categorical) or means and SDs (continuous); SMD < 0.20 indicates adequate balance. “Unknown” cells arise from incomplete HR-derived credential and graduation-year fields in the clinician master, missing at the clinician level independent of AI use; the asymmetry (9.8% control vs. 1.8% AI) reflects more complete records among the more recently credentialed adopter group.

**Table:** 

Part B: Emergency department encounter characteristics			
Characteristics	Usual documentation, *n* = 216,370	Ambient AI, *n* = 42,610	*p* -Value
Patient demographics			
Age, mean ± SD, y	40.7 ± 26.3	42.5 ± 25.5	<0.001
Age, median [IQR], y	39.0 [19–62]	41.0 [22–63]	<0.001
Pediatric (<18 y), *n* (%)	51,779 (23.9)	8246 (19.4)	<0.001
Adult (18–64 y), *n* (%)	115,783 (53.5)	24,310 (57.1)	<0.001
Older adult (≥65 y), *n* (%)	48,808 (22.6)	10,054 (23.6)	0.001
Acuity level (emergency severity index)			
ESI 1: immediate, *n* (%)	4096 (1.9)	405 (1.0)	<0.001
ESI 2: emergent, *n* (%)	40,568 (18.7)	7705 (18.1)	0.02
ESI 3: urgent, *n* (%)	111,274 (51.4)	23,384 (54.9)	<0.001
ESI 4: less urgent, *n* (%)	58,096 (26.9)	10,762 (25.3)	<0.001
ESI 5: nonurgent, *n* (%)	1957 (0.9)	319 (0.7)	0.02
Not triaged/unspecified, *n* (%)	379 (0.2)	35 (0.1)	0.02
Arrival method			
Walk-in/private vehicle, *n* (%)	174,795 (80.8)	35,393 (83.1)	<0.001
EMS/ambulance, *n* (%)	38,590 (17.8)	6659 (15.6)	<0.001
Other/unknown, *n* (%)	2985 (1.4)	558 (1.3)	0.29
ED disposition			
Discharge, *n* (%)	166,502 (77.0)	33,149 (77.8)	0.003
Admit, *n* (%)	26,780 (12.4)	4709 (11.1)	<0.001
Observation, *n* (%)	16,241 (7.5)	3469 (8.1)	0.001
Transfer, *n* (%)	4685 (2.2)	927 (2.2)	0.97
Against medical advice, *n* (%)	1243 (0.6)	280 (0.7)	0.12
Left without being seen, *n* (%)	587 (0.3)	46 (0.1)	<0.001
Expired, *n* (%)	229 (0.1)	20 (0.0)	0.10
Admit level of care (% of admitted/observation encounters)			
Level 1: ICU/critical care, *n* (%)	858 (2.1)	92 (1.2)	0.001
Level 2: intermediate care, *n* (%)	1167 (2.9)	157 (2.1)	0.003
Level 3: progressive care, *n* (%)	6558 (16.1)	1140 (15.0)	0.04
Level 4: acute, *n* (%)	26,587 (65.1)	4909 (64.5)	0.37
Level 5: general, *n* (%)	5628 (13.8)	1306 (17.2)	<0.001
Hospice, *n* (%)	25 (0.1)	7 (0.1)	0.73
Facility level of service			
Level 1: critical, *n* (%)	519 (0.2)	84 (0.2)	0.82
Level 2: high, *n* (%)	6795 (3.1)	1128 (2.6)	0.001
Level 3: moderate, *n* (%)	61,189 (28.3)	11,248 (26.4)	<0.001
Level 4: low, *n* (%)	79,520 (36.8)	17,013 (39.9)	<0.001
Level 5: minor, *n* (%)	45,051 (20.8)	9058 (21.3)	0.10
Unspecified, *n* (%)	23,296 (10.8)	4079 (9.6)	<0.001

Abbreviations: AI, artificial intelligence; ED, emergency department; EMS, emergency medical services; ESI, Emergency Severity Index; ICU, intensive care unit; IQR, interquartile range: SD, standard deviation.

Note: Unless otherwise indicated, data are presented as *n* (%). *p* -Values are approximate; categorical variables compared by chi-square test, continuous variables by Mann–Whitney *U* test. The unit of analysis is the ED encounter (unique visit). A total of 258,980 encounters are included, of which 42,610 (16.4%) had ≥1 ambient AI note and 216,370 (83.6%) had no ambient AI note during the visit. The manuscript reports 315,242 total notes (26,583 AI notes, 8.6%), which is the note-level count; encounter counts differ because a single encounter may generate multiple notes, and AI-adopting clinicians tend to document more frequently. Admit level of care percentages are among admitted and observation encounters only.

The sensitivity analysis was conducted by decomposing the DiD regression model into the four underlying group means: Control Pre-Intervention, Control Post-Intervention, Treatment Pre-Intervention, and Treatment Post-Intervention. Predicted means were calculated directly from the linear regression model, and differences were derived to illustrate the underlying trends. The DiD estimator was then computed as the difference between the pre–post changes in the treatment and control groups. For the within-clinician paired analysis of active editing time, each Mixed-Use clinician’s median active editing time was computed separately for AI-assisted and conventional notes; inference was based on the Wilcoxon signed-rank test on the paired clinician-level differences, with a bootstrap 95% CI (2000 resamples) on the median difference. Encounter rows were deduplicated at the clinician-encounter level and editing-time values were winsorized at the 99th percentile per group ( **Supplementary Table S2** , available in online version only, notes).

To disentangle the effect of the ambient AI tool from the concurrent template change, we leveraged the fact that postimplementation non-AI users documented within the same updated template. A within-period comparison of AI-assisted versus conventional notes in the postimplementation period therefore isolates the incremental effect of the AI tool above and beyond any template-driven efficiency gains, as both groups share the same template exposure.

All analyses were performed using STATA 19 MP (StataCorp LLC, College Station, Texas, United States); the within-clinician editing-time analysis was performed in Python (SciPy 1.11) on Microsoft Fabric.

## Results

### Baseline Characteristics

Primary cohort and analytic window: the primary cohort comprised all ED clinician-authored notes with valid timestamps from May 2024 through June 2025 (315,242 notes; 329 active clinicians). Counts that differed across analyses reflect distinct denominators rather than inconsistent cohorts: 17,780 ambient recording sessions were initiated, of which 13,400 yielded a signed AI-generated note; i.e., some sessions did not result in a final note; 26,583 AI notes constitute the note-level analytic unit and 42,610 encounters included at least one AI note. The longitudinal engagement analysis (Section “Longitudinal Clinician Engagement”) deliberately extends to February to March 2026 and is labeled secondary.

During the study period, 166 of 329 active clinicians (50.5%) used the ambient AI scribe, generating 26,583 AI-generated notes, representing 8.6% of all 315,242 notes authored ( Fig. 1 ). Because a single ED encounter may generate more than one clinical note, encounters associated with AI use ( *n* = 42,610; 16.4% of all encounters) represent a smaller proportion than the total AI-note count. The remaining 216,370 encounters (83.6%) were documented exclusively with usual (non-AI scribe) notes. Clinicians in the intervention arm were demographically and professionally comparable to clinicians who authored usual notes only. Years since graduation were similar across groups (15.9 years [standard deviation, SD: 9.1] control vs. 17.1 years [SD: 8.8] intervention; standardized mean difference [SMD] = 0.13), and distributions of note-volume quartiles were nearly identical, with >50% of both groups falling in the highest-volume quartile ( Table 3 ). Gender and degree composition were likewise balanced (all SMDs < 0.20, Table 3 ). “Unknown” proportions for gender and degree (9.8 control vs. 1.8% AI) reflect incomplete human resources-derived credential fields, which were more complete among the more recently credentialed adopter group and were not differential by AI use.

All clinicians who used AI had a mixed-use of AI and usual notes. Among mixed-use clinicians, the median AI adoption rate was 16.2% (interquartile range [IQR]: 4.2–31.3%), with a median of 286 AI-assisted notes per clinician (IQR: 55–663) out of a median total of 2671 notes (IQR: 1659–3387). This within-clinician variation, where the same clinician generated both AI-assisted and conventional notes, served as the foundation of the study’s design. The absence of any “always AI” users confirms that AI adoption was partial and intermittent rather than a wholesale workflow replacement. The adoption and utilization were robust and incremental 1-year after the study, where 77% of the ED clinicians utilized AI in >40% of their clinical notes (see Sensitivity analysis, Section “Longitudinal Clinician Engagement” and **Supplementary Fig. S1** , available in online version only).

Case-mix analysis demonstrated that encounters documented with the ambient AI scribe were not limited to low-complexity or expedited presentations. Compared with usual-note encounters, AI-documented encounters had similar overall ESI distributions, although with slightly fewer ESI-1 and ESI-2 cases and a modestly higher proportion of ESI-3 encounters (54.9 vs. 51.4%, Table 3 ). Arrival mode patterns were comparable, with AI-note encounters showing only a slightly higher proportion of walk-ins (83.1 vs. 80.8%) and a marginally lower proportion of emergency medical services arrivals (15.6 vs. 17.8%). Disposition profiles were also broadly similar ( Table 3 ).

### Patient Experience

Among 17,780 encounters with an ambient recording session, the mean audio duration was 5.9 min (SD: 3.9; median 5.1; IQR: 3.3–7.5; **Supplementary Table S1** (available in online version only). Of these, 13,400 resulted in an AI-generated note and 4379 were recordings without a generated note.

For “Doctors took the time to listen” ( *n* = 8872 surveys; 123 clinician clusters), AI-assisted encounters had a significantly higher adjusted (for confounders in Table 3 ) probability of a top-box rating (82.0%; 95% CI: 78.7–85.2) than conventional encounters (76.6%; 95% CI, 75.0–78.2), an absolute difference of +5.4 percentage points (95% CI: +2.1 to +8.6; OR: 1.39; 95% CI: 1.12–1.72; *p* = 0.003). While statistically significant, the 5.4-percentage-point absolute difference represents a modest practical effect that should be interpreted in the context of PG score distributions, where top-box rates typically cluster in a narrow range and small absolute shifts can correspond to meaningful percentile changes in institutional benchmarking.

For “Likelihood to Recommend the ED Clinician” ( *n* = 8923 surveys; 123 clinician clusters), the adjusted top-box probability was 77.6% (95% CI: 74.0–81.1) for AI-assisted encounters versus 75.0% (95% CI: 73.5–76.5) for conventional encounters (absolute difference +2.6 percentage points; 95% CI: −1.1 to +6.3; OR: 1.15; 95% CI: 0.93–1.42; *p* = 0.185). This difference was not statistically significant ( **Supplementary Table S5** , available in online version only).

**Fig. 2:**
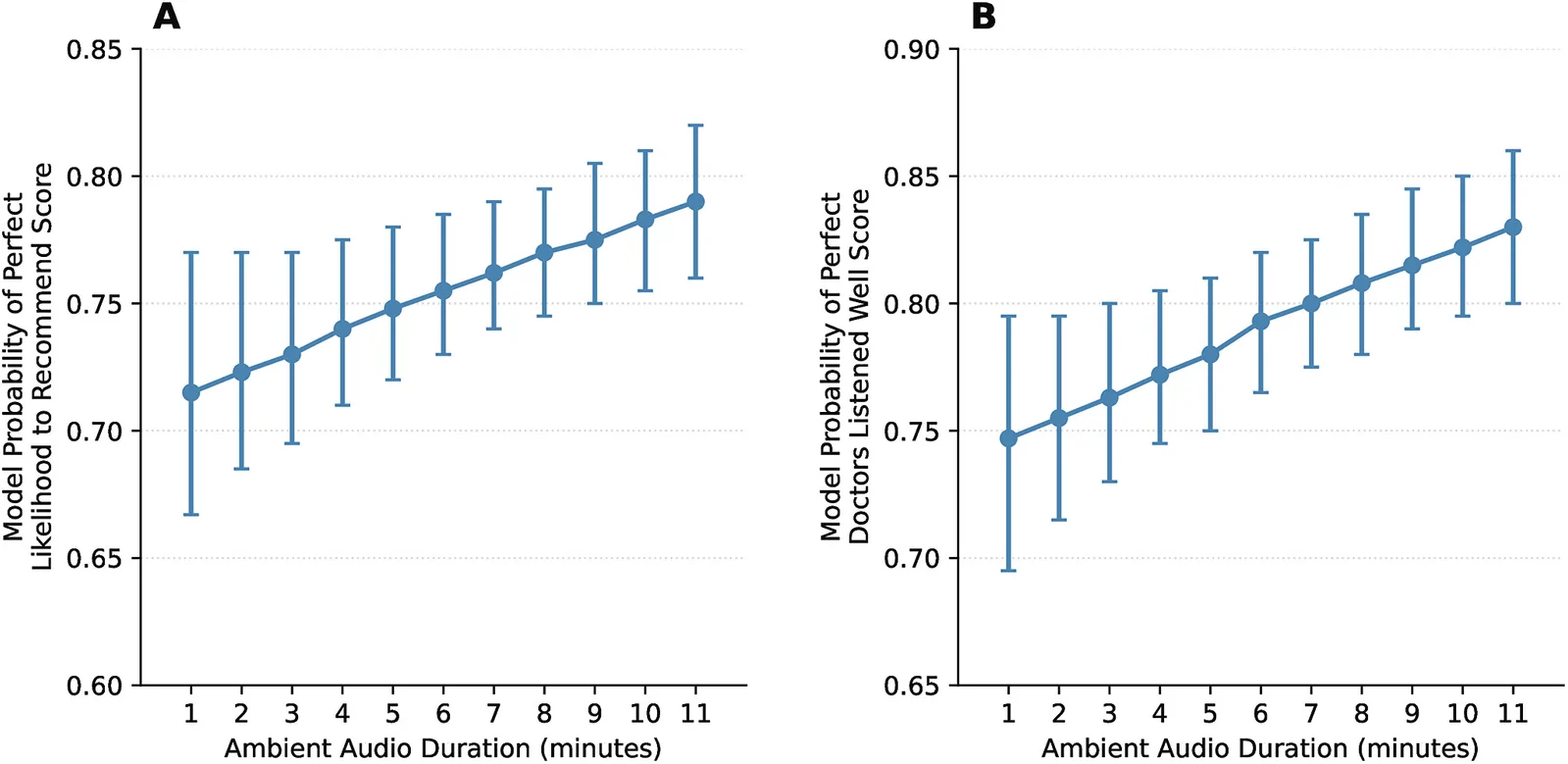
The relationship between top-box scores on “Likelihood to Recommend” and “Doctors took the time to listen” and the duration of the ambient audio recording.

Each additional minute of recorded ambient audio was associated with higher odds of a top-box rating for both “Likelihood to Recommend” (OR: 1.03; 95% CI: 1.00–1.08) and “Doctors listened” (OR: 1.05; 95% CI: 1.01–1.10; Fig. 2 ).

### Documentation Quality, Efficiency, and Timeliness

**Fig. 3:**
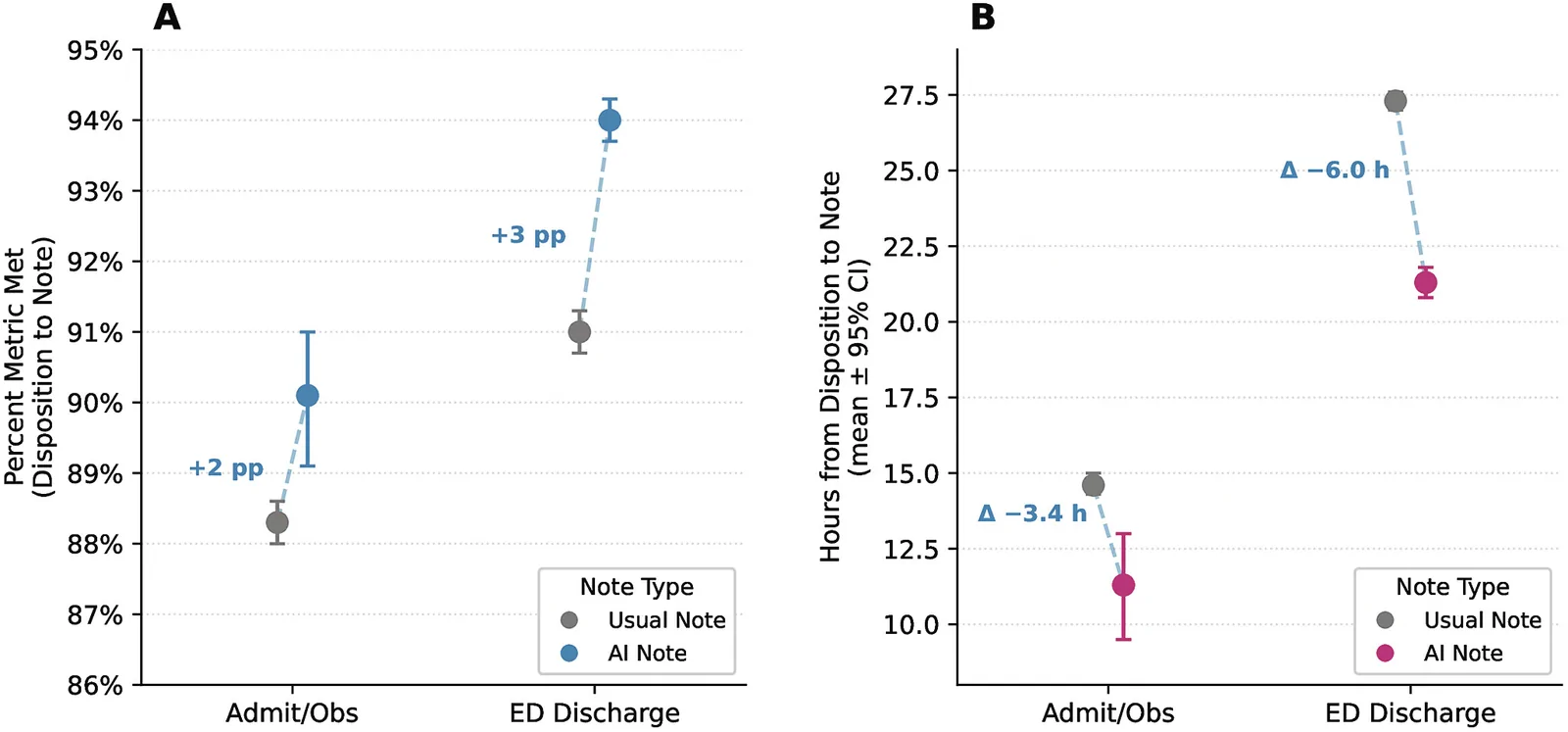
The relationship between use of an AI-generated note and the percent of notes completed within 24 h (admitted/observation) and 72 h (discharge), and the time required for clinicians to complete documentation for patients who were admitted/observed or discharged. AI, artificial intelligence.

Among ED clinical notes, AI use increased the odds of note completion within 24 h by 20% (OR: 1.20; 95% CI: 1.08–1.34), and notes were completed 3.4 h earlier ( Fig. 3 ). Among ED discharges, AI use increased the odds of note completion within 72 h by 56% (OR: 1.56; 95% CI: 1.34–1.67), and notes were completed 6.0 h earlier ( Fig. 3 ).

Compared with usual documentation, clinicians manually typed 722 fewer characters per AI-assisted note and spent 22 fewer seconds in active editing per note in unadjusted note-level comparisons. In the within-clinician paired analysis among 164 Mixed-Use clinicians, active editing time did not differ significantly between AI-assisted and conventional notes (clinician-level median 19.2 vs. 18.4 min; median paired difference +0.17 min; bootstrap 95% CI: −1.00 to +1.55; Wilcoxon signed-rank, *p* = 0.349; **Supplementary Table S2** , available in online version only). These findings indicate that the timeliness benefit reflects earlier initiation of note completion rather than faster editing once the note is opened.

AI-assisted notes were substantially less likely to contain copied-forward characters from prior documentation sources. The predicted probability of copied-forward content was 9.4% for AI-assisted notes compared with 18.3% for conventional notes (absolute difference: −8.9 percentage points; 95% CI: −11.6 to −6.2; OR: 0.46; 95% CI: 0.36–0.60; *p* < 0.001; **Supplementary Table S3** , available in online version only). This finding indicates that ambient AI documentation generates predominantly original clinical narratives derived from the patient encounter rather than recycling text from prior notes, a pattern that may improve note accuracy and reduce the propagation of outdated or erroneous information across the medical record.

### Clinician Cognitive Burden

Pre- and postimplementation NASA-TLX surveys were administered to ED clinicians 2 weeks before and 8 weeks after ambient AI deployment, respectively. Twenty-five of 329 active clinicians (7.6%) responded to the preimplementation survey and 15 (4.6%) to the postimplementation survey. The pre- and postimplementation samples were largely nonoverlapping (anonymous administration precluded individual-level linkage; demographic and role distributions indicated minimal overlap), precluding a paired analysis; independent-samples *t* tests were used. All 15 postimplementation respondents were active users of the ambient AI documentation tool.

The total perceived cognitive burden score, as measured by the NASA-TLX, decreased by 40.2 points postimplementation (pre: 72.1 ± 13.5; post: 31.9 ± 16.9; 95% CI for difference, 30.4–50.1; *t* = 8.30, *p* < 0.001). Substantial reductions were observed across all three subscales: temporal demand decreased by 44.4 points, effort by 39.3 points, and mental demand by 37.1 points (all *p* < 0.001; Table 4 ). The magnitude of these reductions is subject to both selection bias and Hawthorne effect, as discussed in Sections “Discussion and Limitations.”

**Table 4:** Clinician-perceived cognitive burden before and after implementation of ambient artificial intelligence documentation, measured by the NASA Task Load Index.

Cognitive burden measure	Before ( *n* = 25), mean ± SD	After ( *n* = 15), mean ± SD	Mean difference	95% CI	*t*	*p* -Value
NASA-TLX total	72.13 ± 13.52	31.89 ± 16.88	40.24	30.43–50.06	8.30	<0.001
Effort	71.60 ± 17.60	32.33 ± 16.99	39.27	27.78–50.76	6.92	<0.001
Temporal demand	77.40 ± 13.32	33.00 ± 24.91	44.40	32.20–56.60	7.37	<0.001
Mental demand	67.40 ± 20.11	30.33 ± 15.17	37.07	24.87–49.26	6.15	<0.001

Abbreviations: CI, confidence interval; NASA-TLX, National Aeronautics and Space Administration Task Load Index; SD, standard deviation.

Note: Scores range from 0 to 100; higher scores indicate greater perceived cognitive burden. Mean difference = before − after. Comparisons by independent-samples *t* test.

### Sensitivity Analyses

#### Difference-in-Differences Analysis

We decomposed the primary treatment effect using a DiD framework comparing AI adopters versus nonadopters across the pre- and postimplementation periods ( Table 2 ). In the preintervention pilot phase (April–June 2024), two to three clinicians using the AI system completed notes substantially faster than the 248 control clinicians (19.06 vs. 27.67 h, delta = −8.61 h). Following the enterprise rollout beginning March 2025, 166 AI-using clinicians continued to complete notes more quickly than 179 nonusers (21.31 vs. 24.86 h, delta = −3.55 h). The DiD estimator was +5.06 h ( *p* = 0.337), indicating that the pilot cohort’s early advantage attenuated but that the result was not statistically significant. This nonsignificance reflects the extreme sample-size asymmetry inherent to a staggered rollout: the preintervention treatment cell contained only two to three clinicians generating approximately 205 notes, versus 248,634 control notes, a >1,000-fold imbalance that severely limits statistical power to detect a differential trend. IPTW analyses yielded directionally consistent results.

#### Within-Clinician Paired Analyses

To eliminate clinician-level confounding entirely, we conducted within-clinician paired analyses using each clinician as their own control. Among 116 Mixed-Use clinicians with both AI and non-AI notes, AI-assisted notes had higher odds of completion within 72 h (OR: 1.60; 95% CI: 1.21–2.11; *p* = 0.001; predicted probability 94.0% vs. 90.7%; absolute difference +3.3 percentage points; **Supplementary Table S4** , available in online version only). PG top-box Listening scores were also higher for AI-assisted encounters among the same clinicians (82.0% vs. 76.6%; +5.3 percentage points; 95% CI: +2.1 to +8.6; *p* = 0.001; **Supplementary Table S5** (available in online version only). Within-clinician active editing time did not differ between AI-assisted and conventional notes (median paired difference +0.17 min; *p* = 0.349; **Supplementary Table S2** , available in online version only), as reported in Section “Documentation Quality, Efficiency and Timeliness.” These within-clinician designs hold clinician identity constant, providing the most direct evidence that the tool, not clinician characteristics, accounts for the observed documentation and patient experience patterns.

#### Isolating Artificial Intelligence Effect from Template Change

Because the revised documentation template was adopted by both AI users and nonusers during the postimplementation period, the post-only comparison between these groups isolates the incremental AI effect net of any template-related efficiency gains. Among postimplementation encounters, AI-assisted notes were still completed 3.55 h earlier than conventional notes documented within the identical template (21.31 vs. 24.86 h; Table 2 , postintervention column). The within-clinician paired analyses further strengthen this inference: among 116 clinicians who generated both AI and non-AI notes, all using the same updated template, AI-assisted notes had significantly higher odds of on-time completion and higher patient-reported listening scores. Because these within-clinician comparisons hold both clinician identity and template version constant, the observed differences are attributable to the ambient AI tool itself rather than to template redesign.

#### Longitudinal Clinician Engagement

Longitudinal follow-up through February to March 2026 (a deliberate secondary extension beyond the primary analytic window) further addresses the self-selection concern. Among 414 clinicians measured approximately 1-year postdeployment, 202 (48.8%) remained Active Ambient Users and only 20 (4.8%) were classified as dropouts, yielding a 90-day retention rate of approximately 91% among ever-users ( **Supplementary Fig. S1** , available in online version only). Among active users, 77.3% were High Utilizers generating more than 40% of their notes with ambient AI. This degree of sustained, high-intensity integration is inconsistent with a cohort that adopted the tool opportunistically or selectively.

#### Concurrent Workflow Changes and Alternative Outcomes

To evaluate the influence of the concurrent standardized note template, we repeated the primary DiD analysis excluding the 2-month transition period immediately surrounding template rollout. Point estimates and CIs were substantially unchanged ( **Supplementary Table S6** , available in online version only), indicating that template adoption did not materially confound the observed effects. Sensitivity analyses using alternative definitions of documentation timing, including time from discharge decision to note finalization with both continuous and dichotomized thresholds (≤2, ≤4 h), yielded directionally consistent results across all definitions, supporting the robustness of the primary findings.

## Discussion

In a large, high-throughput emergency care network comprising multiple ED sites within a large integrated health system, encompassing 14 EDs sharing a common EHR, deployment of an ambient AI documentation system was steadily adopted, consistently utilized ( **Supplementary Fig. S1** , available in online version only) and associated with measurable improvements across patient-reported experience, cognitive burden, and timeliness of the clinical record.

These findings supplement a growing literature showing that ambient AI documentation can reduce documentation burden and improve clinician experience. 10 11 13 14 15 Prior work in ambulatory care demonstrated reduced after-hours documentation and lower documentation burden with ambient AI scribes, while more recent multicenter and randomized studies have reported improvements in documentation efficiency, burnout-related outcomes, cognitive task load, and perceived ability to focus on patients. 10 11 13 14 15

Across both the DiD sensitivity analysis ( Table 2 ) and the within-clinician paired analysis ( **Supplementary Table S2** , available in online version only), we did not find evidence of reduced active editing time per note, indicating that ambient AI may *not* substantially reduce the final revision burden once a draft is generated versus a robust template with smart form elements.

Rather, the more important signal was a *pragmatic ED quality metric* : earlier chart closure and note completion ( Fig. 3 ). In the ED, where clinicians manage rapid task switching and high patient turnover, earlier closure of charts may reduce the backlog of unfinished documentation, decrease after-hours charting (pajama time), and lessen the anxiety and cognitive burden associated with incomplete notes. This is a meaningful finding for clinicians’ well-being, since documentation performed later in the day or outside scheduled work has been associated with greater EHR burden and burnout. Earlier note finalization may also improve handoffs, support more contemporaneous communication, facilitate coding and billing workflows, and enhance the availability of the clinical record for downstream teams. In this context, charts closed hours earlier may be more operationally and professionally meaningful than single-digit minute differences in editing time alone.

Our study adds large-scale, multisite ED-specific evidence to the corpus of existing literature. This makes it important in our opinion, since emergency medicine evidence cannot be extrapolated from ambulatory care literature, given substantial differences in pace, patient turnover, acuity, interruption burden, and decisional compression. A newly published ED study by Preiksaitis et al similarly found real-world adoption of ambient AI scribes in emergency care and reported differences in documentation time and note characteristics using EHR audit logs, providing an external ED comparator that supports the plausibility and generalizability of our findings. 13

Accordingly, our results are best framed as among the first large-scale, multisite ED evaluations showing that benefits previously observed in ambulatory settings can also be realized in the high-acuity, time-pressured ED. The consistency in directional effects across ambulatory, multicenter, randomized, and now ED-based studies strengthens the inference that ambient AI documentation is associated with meaningful workflow change rather than isolated local effects. 10 11 13 14 15

### Patterns of Intermittent Adoption

Adopters used the tool in a minority of their notes even after sustained access (median adoption rate: 16.2%), and case complexity did not account for this selectivity. Several nonmutually exclusive mechanisms may contribute. First, clinicians may reserve ambient capture for encounters with clear, narrative-driven histories and avoid it where conversation is fragmented, multilingual, interrupted, or dominated by nonverbal assessment. Second, ambient capture depends on a recordable spoken encounter; resuscitations, intoxicated or noncommunicative patients, sensitive disclosures, and rapid procedural care are poorly suited to it. Third, workflow friction device handling, login, ambient-mode activation, and the need to review and edit drafts, may make manual documentation faster for short or templated visits. Fourth, habit and trust calibration evolve gradually; the longitudinal rise to high utilization (Section “Longitudinal Clinician Engagement”) is consistent with selective early use that broadens as clinicians learn which encounters the tool serves well. These hypotheses are not directly testable in the present dataset and motivate prospective study of encounter-level adoption drivers.

### Patient Experience

The finding that AI-assisted encounters had a higher top-box listening score, 82.0% versus 76.6%, warrants careful interpretation. Although statistically significant, the practical importance depends on institutional context, because patient experience scores often cluster within narrow ranges, and modest absolute shifts may translate into meaningful percentile movement. At the same time, the absence of a significant difference in “Likelihood to Recommend” suggests that the observed effect may be domain-specific, reflecting improved *perceived* attentiveness rather than a broad change in overall care perception. We cannot, however, undervalue the *human need of empathy:* to be listened to and understood in a time of a personal health crisis.

This interpretation is consistent with the latest ambient scribe documentation literature. In a large multicenter quality improvement study, ambient AI use was associated with a greater clinician-reported ability to provide *undivided attention* to patients. In parallel, studies of patient attitudes and consent processes suggest that many patients perceive ambient documentation as potentially beneficial when it allows clinicians to focus more fully on the encounter, but that trust, education, and consent remain central mediators of acceptance. 15 16 17

Several local factors could still influence PG scores independently of ambient AI, including staffing levels, crowding, throughput, site leadership, and concurrent communication-focused initiatives. Although the 14-site design reduces the likelihood that the result is entirely health-system specific, residual confounding cannot be excluded. The observed *dose–response relationship* between audio-recording duration and top-box listening scores and Likelihood to Recommend ( Fig. 2 ) provides supportive, though not definitive, evidence that the ambient AI-mediated workflow itself may contribute to the effect. Because ambient audio duration does not perfectly represent bedside interaction time, especially when pauses or off-mic discussion occur, it is best interpreted as a *surrogate* for AI-captured clinician–patient conversation rather than a literal measure of time spent in direct interaction.

### Documentation Efficiency and Quality

The reduction in copied-forward content is one of the most notable findings of this study. Copy-forward practices have long been associated with note bloat, duplication of outdated information, reduced readability, and potential medicolegal and patient safety risks. In emergency medicine specifically, *note bloat* is especially problematic because clinicians often need to extract time-sensitive, high-yield information under conditions of diagnostic uncertainty and high throughput. From that perspective, the lower prevalence of copied-forward text in AI-assisted notes suggests a potential qualitative advantage of ambient documentation: notes may be more encounter-specific and less dependent on recycled templates. 18 19

Of note, greater encounter specificity should not be conflated with verified documentation quality. Our study did not directly assess factual accuracy, completeness, internal consistency, omission errors, hallucinated content, or downstream effects on decision-making. This is an important limitation because recent safety-focused work has shown that AI-enabled scribe products can generate documentation errors that may carry patient safety implications if not detected during clinician review. Likewise, recent evidence synthesis and commentary in this area emphasize that implementation benefits should not outpace rigorous assessment of validity, error patterns, transportability, and governance. 20 21 22

### Clinician Cognitive Burden

The observed reduction in NASA-TLX scores should be interpreted cautiously, particularly given the limitations of subjective workload measurement and the possibility of expectancy effects. However, the direction of the finding aligns with a broader literature suggesting that ambient AI can reduce note-related cognitive burden. In multicenter and randomized studies, ambient AI use has been associated with lower cognitive task load, less documentation burden, and less time spent documenting after hours. The convergence of these findings with our objective metrics, including fewer typed characters, earlier note completion, and higher on-time completion, strengthens the argument that the intervention altered workflow in ways clinicians experienced as less cognitively taxing. 11 14 15 23 24 25

That said, ambient AI may not simply remove cognitive work. Rather, it may redistribute it. Manual composition and clerical data entry are reduced, but clinicians assume a more supervisory role in which they must rapidly detect subtle inaccuracies, omissions, or contextual distortions in AI-generated drafts. Thus, one form of workload may be reduced while another, verification and exception monitoring, becomes more prominent. This distinction is important for interpreting workload measures and for designing future studies that separately assess writing burden, editing burden, vigilance burden, and automation bias 7 8 9 21

### Sensitivity Analyses and Self-Selection

The nonsignificant DiDs result deserves explicit acknowledgment. Given the extreme imbalance in the preintervention treatment cell, the DiDs framework was likely underpowered to detect a differential trend of realistic magnitude. In that context, the within-clinician paired analyses are arguably more informative, because they control for stable clinician-level characteristics by design. The fact that the same clinicians produced earlier, timelier, and less copy-forward-dependent notes during AI use supports the interpretation that the observed benefits were not solely due to baseline differences between adopters and nonadopters.

### Sociotechnical Implications

From a sociotechnical perspective, ambient AI documentation represents more than a simple efficiency tool. It changes authorship, workflow, and accountability. The clinician is no longer the sole primary generator of the clinical narrative, but instead *becomes a reviewer, editor, and legal signatory of a machine-assisted draft* . This shift has implications for trust, liability, professional identity, and clinical reasoning. Recent work on informed consent shows that patients and clinicians view transparency around recording, data handling, and the purpose of ambient documentation as important to maintaining trust. At the same time, safety studies indicate that *the clinician review step is not optional but essential* , because draft notes may contain clinically meaningful errors. 16 21

Accordingly, long-term evaluation of ambient AI should move beyond efficiency metrics alone. Future work should examine note accuracy, omission and hallucination rates, specialty-specific error modes, patient trust, legal responsibility, and the effects of prolonged reliance on AI-generated drafts on clinical reasoning and documentation skills. Recent evidence syntheses highlight that the field is expanding quickly, but that much of the literature remains in early phase, heterogeneous, and concentrated outside emergency medicine. Our findings therefore support cautious optimism: ambient AI documentation appears promising in the ED, but its long-term value will depend on demonstrating not only efficiency and user satisfaction, but also reliability, safety, and durable patient-centered implementation. 20 21 22

## Limitations

This study has several limitations that should be considered when interpreting the findings.

First, the observational design remains vulnerable to self-selection bias. Clinicians who adopted ambient AI may differ from nonadopters in baseline efficiency, motivation, comfort with technology, or documentation style. We attempted to reduce this risk through several complementary strategies: staggered deployment that limited discretion over initial exposure timing, within-clinician paired analyses that used each clinician as their own control, and IPTW. These approaches yielded directionally consistent results, which strengthens confidence in the observed associations. Nonetheless, residual bias cannot be excluded because clinicians could still choose whether to activate the tool within their exposure window, and adoption behavior may have tracked with unmeasured workflow, staffing, or departmental factors.

Second, a standardized note template redesign affecting the HPI, physical examination, and assessment and plan sections was introduced concurrently with ambient AI deployment, creating a potential confound for efficiency outcomes. Because the revised template was implemented system-wide and was used by both AI and non-AI documentation in the postimplementation period, the post-only between-group comparisons and within-clinician paired analyses are partially protected from this source of bias. The same clinician, using the same template, still demonstrated differences in timeliness and patient experience according to AI use. By contrast, the pre-to-post DiDs analysis is more susceptible to confounding because the template change and the AI rollout occurred in overlapping time periods. The null DiDs result should therefore be interpreted cautiously, and future evaluations with staggered template and AI rollouts would allow cleaner causal separation of these effects.

Third, the NASA-TLX findings should be interpreted cautiously. The surveys were completed by small, largely nonoverlapping clinician samples before and after implementation, which required an independent-samples comparison rather than a true paired design. The postimplementation respondents were all active users of the ambient AI tool and therefore represented a self-selected subgroup that may have been more likely to report favorable experiences. In addition, the survey was unblinded and conducted in the context of a visible institutional rollout, which increases the risk of expectancy effects and novelty-related response bias. For these reasons, the observed reduction in subjective workload is best interpreted as suggestive rather than definitive. Importantly, the direction of the NASA-TLX findings was supported by objective workflow measures, which offers independent corroboration that the intervention was associated with measurable changes in documentation practice.

Fourth, the patient experience analysis is limited by survey nonresponse and by the structure of ED-specific PG sampling. Although the number of returned surveys for the listening item was substantial, respondents still represented only a subset of total encounters, and patients admitted from the ED are commonly excluded from these surveys. As a result, the patient experience findings may generalize more directly to discharged patients than to the full ED population. In addition, our emphasis on selected physician-specific items raises the possibility of item-level reporting bias. Future studies should examine the broader portfolio of patient experience measures and should ideally incorporate complementary qualitative assessment of patient perceptions, trust, and consent, especially as newer literature suggests that acceptance of ambient documentation depends in part on how the technology is explained and implemented with patients. 16 17

Fifth, this study did not directly evaluate the factual accuracy, completeness, internal consistency, or clinical quality of AI-generated documentation, nor did it assess downstream effects on coding, billing, utilization, or clinical decision-making. This remains a major evidence gap. Recent work has shown that AI-enabled scribe systems can produce omission errors and other clinically meaningful inaccuracies, while rapid evidence syntheses continue to conclude that the real-world literature is promising but still sparse and methodologically heterogeneous. 21 22 No large-scale validation study in the ED setting has yet established whether ambient AI documentation is consistently accurate across varying acuity, complexity, and communication conditions.

Finally, the study was conducted within a single integrated health system using a shared EHR and common administrative infrastructure across 14 EDs. This multisite structure improves internal consistency and provides replication across diverse operational contexts, but it does not establish external generalizability to other health systems, EHR platforms, ambient AI products, staffing models, or clinical specialties. Additional multicenter studies, including settings outside emergency medicine and across different implementation architectures, will be necessary to determine the portability of these findings.

Despite these limitations, the convergence of objective timeliness measures, reduced copy-forward behavior, selected patient experience improvements, and supportive subjective workload findings across multiple analytic approaches provides a coherent signal that ambient AI documentation may offer meaningful benefits in high-throughput emergency care settings.

## Conclusion

Ambient AI-assisted documentation was associated with higher patient-reported listening, reduced documentation burden, less copied-forward content, and faster note completion. These systems may help enhance clinical presence by improving the timeliness of the medical record, a rare alignment of patient experience, clinician well-being, and operational performance, whereas they add the new responsibility of reviewing, editing, and legally signing machine-assisted draft notes.

**Clinical Relevance Statement**

This study demonstrates that ambient AI documentation tools can reduce clinician workload and improve documentation efficiency across 14 ED sites in a single integrated health system. For practitioners, this technology offers a practical way to decrease clerical burden, allowing more time for direct patient care. For health care organizations, these findings highlight a scalable intervention to improve operational throughput and support clinician well-being.

**Multiple-Choice Questions**

1. In this study, the implementation of an ambient AI documentation tool was associated with which of the following efficiency outcomes?
  - Increased characters typed per note.
  - Longer time spent editing notes.
  - Faster note completion time for both admitted and discharged patients.
  - No meaningful change in documentation timeliness.
  **Correct Answer** : The correct answer is option c. The study found that documentation was completed 3.4 h earlier for admitted patients and 6.0 h earlier for discharged patients. The AI tool was associated with fewer characters typed and earlier on-time note completion, whereas active editing time per note was not significantly altered.
2. According to the NASA-TLX survey results, what was the most prominent effect associated with ambient AI implementation in this cohort?
  - It increased the mental demand of documentation.
  - It had no measurable effect on perceived workload.
  - It slightly increased feelings of being rushed.
  - It was associated with a substantial decrease in total perceived cognitive burden.
  **Correct Answer** : The correct answer is option d. The results showed a decrease of 40.2 points in the NASA-TLX total workload score after implementation, accompanied by reductions in mental demand, temporal demand, and effort subscales. These findings should be interpreted cautiously given the small samples, self-selection of postimplementation respondents, and possible Hawthorne effect.
3. When evaluating an ambient AI documentation tool deployed via a staggered rollout, which design feature most directly mitigates self-selection bias from voluntary adoption?
  - A before-after analysis restricted to adopters.
  - A cross-sectional clinician satisfaction survey.
  - A difference-in-differences analysis with clinician-level clustering and within-clinician paired comparisons.
  - A single-arm time-series without control clinicians.
  **Correct Answer** : The correct answer is option c. DiD compares adopters and nonadopters across pre- and postimplementation periods, whereas clinician-level clustering accounts for repeated measures within clinicians. Combined with within-clinician paired analyses, in which each clinician serves as their own control, this approach removes time-invariant clinician characteristics and is therefore the most informative design for addressing self-selection in observational ambient-AI studies.

## Declaration of GenAI Use

During the writing process of this paper, the authors used Claude (Anthropic Inc., San Francisco, California, United States) to assist with manuscript drafting, language editing for clarity and readability, structuring the point-by-point response to reviewers, and formatting of supplemental materials. The authors reviewed and edited all text and take full responsibility for the content of the paper.

## Supplementary Material

Ergänzendes Material Supplementary Material
