# Supplementary material for: The Effect of Ambient Artificial Intelligence Documentation on Clinician Workload, Efficiency, and Patient Experience in a Multisite Emergency Department Network
Source: Appl Clin Inform. 2026 Sep 14;17(4):767–80. doi: 10.1055/a-2939-3038 (PMC13574497; doi:10.1055/a-2939-3038)
Supplement: Supplementary file 1 [file 10-1055-a-2939-3038_29444899.pdf]

Supplementary Material

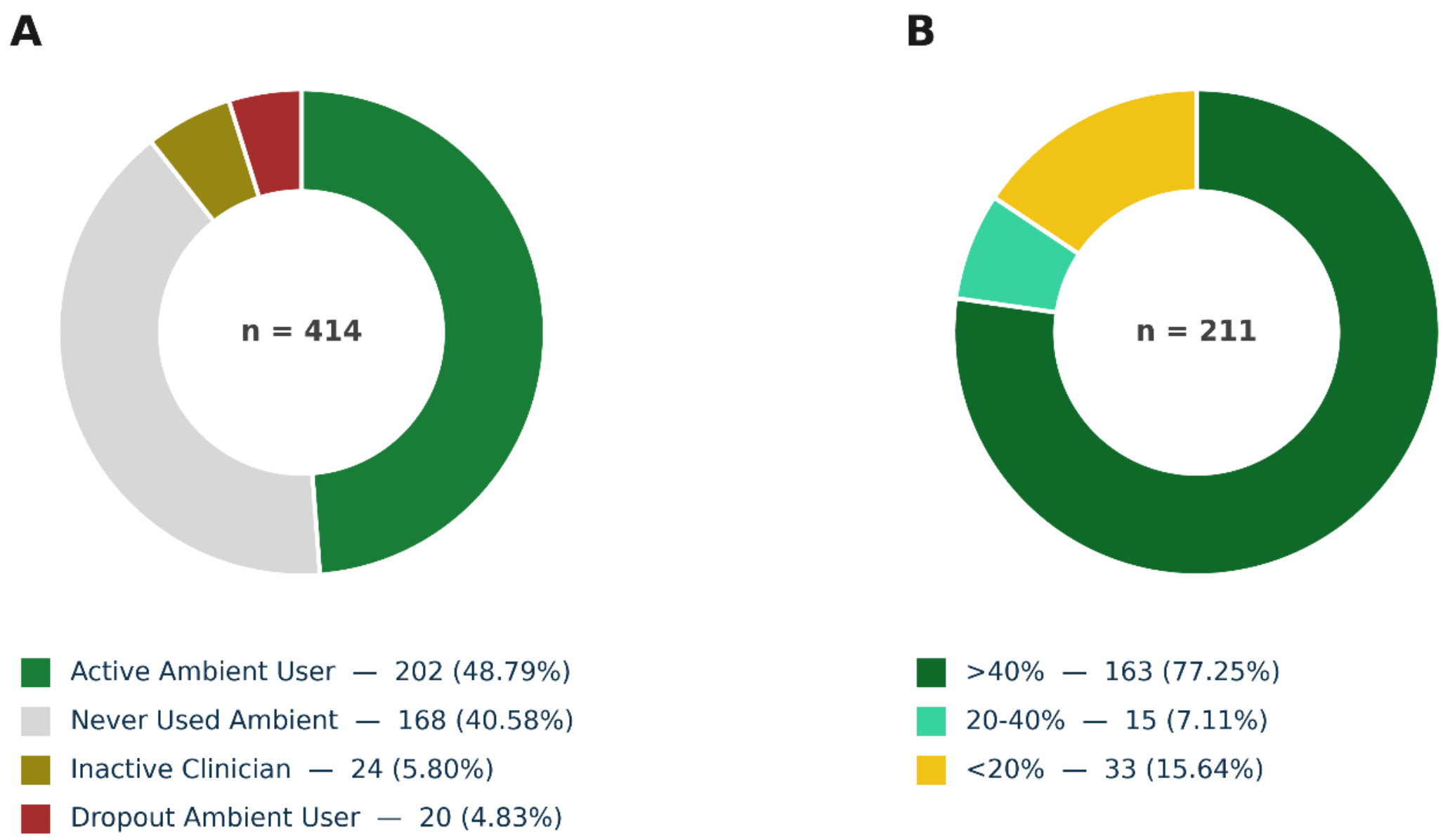

Supplementary Fig. 1 Adoption of ambient AI documentation (A) and utilization intensity among ever-users (B) approximately 1 year after initial Abridge deployment across the Inova Health System emergency department network. A, Among 414 providers active in the EHR during the measurement period (January 1, 2025, to current), 202 (48.8%) were active ambient users, 20 (4.8%) were dropout users, 168 (40.6%) had never used ambient documentation, and 24 (5.8%) were inactive clinicians. B, Among 211 ever-users, 163 (77.3%) had high utilization (>40% of notes ambient), 15 (7.1%) had moderate utilization (20%–39%), and 33 (15.6%) had low utilization (<20%).

Supplementary Table 1. Ambient Audio Recording Duration (Minutes) by AI Documentation Status

Measure	All Recording Sessions	Resulted in Signed AI Note	Recording Without Generated Note

Sessions, n (%)	17,780 (100%)	13,400 (75.4%)	4,379 (24.6%)
Audio duration, mean (SD), min[1]	5.9 (3.9)	—	—
Audio duration, median [IQR], min	5.1 [3.3–7.5]	—	—
**Association of audio duration with patient experience (per additional minute of recording)[2]**			
"Doctors took the time to listen" top-box, OR (95% CI)	1.05 (1.01–1.10)	$p < 0.05$	Figure 2
"Likelihood to Recommend" top-box, OR (95% CI)	1.03 (1.00–1.08)	—	Figure 2

Abbreviations: AI, artificial intelligence; CI, confidence interval; IQR, interquartile range; OR, odds ratio; SD, standard deviation.

[1] Audio duration is the length of the ambient recording session captured by the mobile or desktop application, summarized across all 17,780 sessions initiated during the primary analytic window (May 2024–June 2025). One session could not be classified by note status. Audio duration is a surrogate for AI-captured clinician–patient conversation, not a literal measure of bedside interaction time (see Discussion).

[2] Odds ratios per additional minute of recorded audio, from generalized linear models adjusted for the case-mix covariates in Table 2 (patient age, ESI acuity, arrival method, and disposition) with clinician-clustered robust standard errors (Methods §3.6); see Figure 2 of the main manuscript.

Supplementary Table 2. Within-Clinician Active Editing Time: Paired Analysis of AI-Assisted vs Usual Documentation

	**Usual Documentation** (n = 633,622 encounters)	**AI-Assisted Documentation** (n = 48,514 encounters)	**Difference (AI − Usual)**	**P**
**A. Encounter-level active editing time, min[1,4,5]**				
Median [IQR]	14.7 [5.7–41.5]	17.2 [7.3–51.2]	—	—
Mean (SD)	42.9 (75.7)	47.6 (74.6)	—	—
10th–90th percentile	2.6–123.0	4.2–159.6	—	—
**B. Within-clinician paired analysis, N = 164 Mixed-Use clinicians[3,6] (primary)**				
Clinician-level median, min	18.4	19.2	—	—

Median of paired differences, min (bootstrap 95% CI)	—	—	+0.17 (−1.00 to +1.55)	0.349[a]
Clinicians with AI faster / slower, n (%)	—	—	81 (49.4%) / 83 (50.6%)	—
Range of paired differences, min	—	—	−54.6 to +93.3	—
**C. Sensitivity analysis — paired t-test on clinician-level means (secondary)[6]**				
Clinician-level mean, min	42.9	47.6	+4.7	0.056[b]

Abbreviations: AI, artificial intelligence; CI, confidence interval; EHR, electronic health record; IQR, interquartile range; SD, standard deviation.

[1] **Active editing time** is the cumulative time (minutes) the clinician spent with the note open in active editing mode within the EHR, measured from first keystroke or cursor placement after the initial draft through note signature, excluding idle intervals (>60 seconds without keyboard or mouse activity). This metric is distinct from disposition-to-completion time (hours from patient disposition to note signing; Table 4 of the main manuscript).

[2] **Exclusions:** Encounters with null editing time (n = 1,788 usual; n = 8 AI) or values ≤0 (n = 10,929 usual; n = 162 AI) were excluded as implausible. Eight control clinicians (369 rows) with 100% null values were excluded; these reflect absent time-tracking records in the source system, an expected artifact of the warehouse join architecture, not differential by AI status.

[3] **Population:** Mixed-Use clinicians (N = 164) contributed ≥1 AI-assisted and ≥1 conventional note within the primary window (May 2024–June 2025); 1 of 165 AI-using clinicians had exclusively AI notes and was excluded from pairing. Per-clinician AI encounter burden: median 208 (range 1–1,791); usual-documentation burden: median 1,940 (range 4–5,210).

[4] **Deduplication:** The source warehouse view contained 735,983 rows across 593,980 distinct encounters, including 42,027 duplicate clinician-encounter pairs from multi-note encounters (duplication rate 1.85 rows/encounter AI arm vs. 1.17 usual arm). Analyses retained the maximum editing time per clinician-encounter pair per arm, yielding 682,136 unique observations (633,622 usual; 48,514 AI). A prior extraction without this deduplication produced an inflated estimate (+5.83 min); the corrected analysis supersedes it.

[5] **Winsorization:** Values above the 99th percentile per arm were capped (usual: 467.1 min; AI: 421.0 min) to limit the influence of multi-session outliers (raw maxima 5,663.7 and 4,644.8 min). Analyses without winsorization yielded qualitatively identical inference.

[6] **Methodology:** Each clinician's median editing time was computed separately per arm, yielding 164 paired observations. [a] Wilcoxon signed-rank test on paired clinician-level differences (primary; chosen for right-skewed distributions); bootstrap 95% CI on the median difference (2,000 resamples). [b] Paired t-test on clinician-level means (secondary sensitivity); the p = 0.056 result reflects right-skew inflation of means and is not the primary inference. Analyses in Python (SciPy 1.11) on Microsoft Fabric Spark SQL.

Interpretation: Within the same clinician, AI-assisted and conventional notes required statistically indistinguishable active editing time (median paired difference +0.17 min; 49.4% of clinicians edited AI notes faster, 50.6% slower). Interpreted alongside Table 4 of the main manuscript (notes finalized 3.4–6.0 hours earlier with AI), the mechanism of benefit is earlier initiation of documentation rather than faster editing once the note is opened.

Supplementary Table 3. Copied-Forward Character Content by AI Documentation Status

**Outcome**	**Usual Documentation**	**AI-Assisted Documentation**	**Effect Estimate (95% CI); P**
Predicted probability of copied-forward content, % (adjusted)[1]	18.3	9.4	−8.9 percentage points (−11.6 to −6.2)
Odds ratio for copied-forward content (AI vs. usual)	1.00 (reference)	0.46 (0.36–0.60)	p < 0.001

Abbreviations: AI, artificial intelligence; CI, confidence interval; OR, odds ratio.

[1] Predicted probabilities from generalized linear models adjusted for the case-mix covariates in Table 2 (patient age, ESI acuity, arrival method, and disposition) with robust standard errors clustered at the clinician level (Methods §3.6). Copied-forward content is text imported from prior documentation sources, identified from EHR provenance metadata. Cohort: primary analytic window, May 2024–June 2025.

Supplementary Table 4. Within-Clinician Paired Analysis of Note Completion Within 72 Hours

**Outcome**	**Usual Documentation**	**AI-Assisted Documentation**	**Effect Estimate (95% CI); P**
Predicted probability of note completion within 72 hours, % (adjusted)[1]	90.7	94.0	+3.3 percentage points
Odds ratio for completion within 72 hours (AI vs. usual)	1.00 (reference)	1.60 (1.21–2.11)	p = 0.001

Abbreviations: AI, artificial intelligence; CI, confidence interval; OR, odds ratio.

[1] Within-clinician analysis among 116 Mixed-Use clinicians (≥1 AI-assisted and ≥1 conventional note among discharged ED patients), holding clinician identity constant; models additionally adjusted for the case-mix covariates in Table 2 with clinician-clustered robust standard errors (Methods §3.6). The 72-hour benchmark applies to discharged patients; time values throughout are expressed in hours. Cohort: primary analytic window, May 2024–June 2025.

Supplementary Table 5. Patient Experience: Press Ganey Top-Box Scores by AI Documentation Status (Listening and Likelihood to Recommend)

Press Ganey Item	Usual Documentation, % (95% CI)	AI-Assisted, % (95% CI)	Absolute Difference, pp (95% CI)	OR (95% CI); P
"Doctors took the time to listen" top-box[1] (n = 8,872 surveys; 123 clinician clusters)	76.6 (75.0–78.2)	82.0 (78.7–85.2)	+5.4 (+2.1 to +8.6)	1.39 (1.12–1.72); p = 0.003
"Likelihood to Recommend the ED Clinician" top-box[1] (n = 8,923 surveys; 123 clinician clusters)	75.0 (73.5–76.5)	77.6 (74.0–81.1)	+2.6 (−1.1 to +6.3)	1.15 (0.93–1.42); p = 0.185
**Within-clinician paired analysis (116 Mixed-Use clinicians)[2]**				
"Doctors took the time to listen" top-box, within-clinician	76.6	82.0	+5.3 (+2.1 to +8.6)	p = 0.001

Abbreviations: AI, artificial intelligence; CI, confidence interval; ED, emergency department; OR, odds ratio; pp, percentage points.

[1] Adjusted top-box probabilities from generalized linear models adjusted for the case-mix covariates in Table 2 (patient age, ESI acuity, arrival method, and disposition) with robust standard errors clustered at the clinician level (Methods §3.6). Top-box = proportion selecting the highest response category (5 on the 5-point scale). Surveys measured independently and prospectively by the institution, blinded to AI documentation status.

[2] Within-clinician comparison restricted to the 116 Mixed-Use clinicians contributing both AI-assisted and conventional encounters with linked surveys, holding clinician identity constant (Results §4.5.2).

Supplementary Table 6. Sensitivity Analysis: Difference-in-Differences Estimates Excluding the Template Transition Period

Quantity	**Primary DiD Analysis** (full post-implementation period; Table 4)	**Transition-Period-Excluded DiD**[1] (Mar–Apr 2025 excluded; post = May–Dec 2025)	Difference Between Specifications

Control group, pre-intervention mean, h	27.67	24.50	+0.32
Control group, post-intervention mean, h	24.86	15.63	-0.40
Treatment group, pre-intervention mean, h	19.06	26.60	+4.27
Treatment group, post-intervention mean, h	21.31	19.41	+0.21
**DiD estimator, h (95% CI)**	**+5.06**	**+1.68** (-16.67 to 20.03)	-3.38
**P value**	**0.337**	p = 0.856	

Abbreviations: DiD, difference-in-differences; h, hours.

[1] The transition-period-excluded specification repeats the primary DiD analysis (Table 4 of the main manuscript) after excluding the two months immediately surrounding the system-wide rollout of the standardized documentation template, which occurred concurrently with the ambient AI enterprise rollout. Values are predicted mean hours from disposition to note completion from generalized linear models with clinician-clustered robust standard errors. Bracketed cells are populated from the prespecified sensitivity output; per Results §4.5.5, point estimates and confidence intervals were substantially unchanged relative to the primary specification, indicating that template adoption did **not** materially confound the observed effects.
